# Oxidative Stress and Apoptotic Responses Elicited by *Nostoc*-Synthesized Silver Nanoparticles against Different Cancer Cell Lines

**DOI:** 10.3390/cancers12082099

**Published:** 2020-07-28

**Authors:** Reham Samir Hamida, Gadah Albasher, Mashael Mohammed Bin-Meferij

**Affiliations:** 1 Molecular Biology Unit, Department of Zoology, Faculty of Science, Alexandria University, Alexandria 21500, Egypt; 2 Zoology Department, College of Science, King Saud University, Riyadh 11543, Saudi Arabia; galbeshr3@gmail.com; 3 Department of Biology, College of Science, Princess Nourah bint Abdulrahman University, Riyadh 11543, Saudi Arabia

**Keywords:** cancer, nanoparticle, apoptotic pathway, mTOR signaling pathway, ultrastructural change, p53, *Nostoc* sp.

## Abstract

Green nanoparticles represent a revolution in bionanotechnology, providing opportunities to fight life-threatening diseases, such as cancer, with less risk to the environment and to human health. Here, for the first time, we systematically investigated the anticancer activity and possible mechanism of novel silver nanoparticles (N-SNPs) synthesized by *Nostoc* Bahar M against the MCF-7 breast cancer cells, HCT-116 colorectal adenocarcinoma cells, and HepG2 liver cancer cells, using cell viability assays, morphological characterization with inverted light and transmission electron microscopy, antioxidants and enzymes (glutathione peroxidase (GPx), glutathione (GSH), adenosine triphosphatase (ATPase), and lactate dehydrogenase (LDH)), and western blotting (protein kinase B (Akt), phosphorylated-Akt (p-Akt), mammalian target of rapamycin (mTOR), B-cell lymphoma 2 (Bcl-2), tumor suppressor (p53), and caspase 3). N-SNPs decreased the viability of MCF-7, HCT-116, and HepG2 cells, with half-maximal inhibitory concentrations of 54, 56, and 80 µg/mL, respectively. They also significantly increased LDH leakage, enhanced oxidative stress via effects on antioxidative markers, and caused metabolic stress by significantly decreasing ATPase levels. N-SNPs caused extensive ultrastructural alterations in cell and nuclear structures, as well as in various organelles. Furthermore, N-SNPs triggered apoptosis via the activation of caspase 3 and p53, and suppressed the mTOR signaling pathway via downregulating apoptosis-evading proteins in MCF-7, HCT-116, and HepG2 cells. Ultrastructural analysis, together with biochemical and molecular analyses, revealed that N-SNPs enhanced apoptosis via the induction of oxidative stress and/or through direct interactions with cellular structures in all tested cells. The cytotoxicity of *Nostoc*-mediated SNPs represents a new strategy for cancer treatment via targeting various cell death pathways. However, the potential of N-SNPs to be usable and biocompatible anticancer drug will depend on their toxicity against normal cells.

## 1. Introduction

Cancer has a major impact on human life today, owing to enormous changes in lifestyle, and it is the second leading cause of death worldwide [1]. Normal cells avoid undergoing tumorigenesis through the regulation of cellular mechanisms underlying vital processes, such as proliferation and cellular growth; however, any deviations in these processes may result in cancer [2]. Cancer cells have the ability to evade apoptosis via controlling the expression of certain genes; that is, the upregulation of the expression of genes that favor survival and proliferation, and downregulation of genes that are responsible for the regulation of cell death pathways [3]. Conventional anticancer therapies, such as chemotherapeutic drugs, radiation, and surgery, are successful to some extent, but their use is limited by serious adverse effects and poor diagnosis, and by the potential for cancer cells to develop resistance to chemotherapeutic drugs [4].

Thus, there is a need for new and more effective therapies to fight this disease. Nanotechnology has been used to develop next-generation platforms for cancer diagnosis, therapy, and management [5,6,7]. The nano-revolution affords opportunities for researchers to create, improve, and develop nanoparticle (NP)-based products for use in many medical domains, including pharmaceutical applications, drug delivery, bioimaging, biolabeling, diagnostics, and medical nanodevices [8]. Nanotechnology also allows to us to clearly understand the interactions between nanoscale materials or particles and living cells, in order to create medical solutions to various serious diseases [9]. Furthermore, progress in materials and protein technology has led to a new nanoscale targeting method that may increase the safety and efficiency of therapies for cancer patients [10]. 

Unlike small-molecule drugs, NPs are distinguished by unique physicochemical features, including a large surface area to volume ratio, permitting these particles to easily penetrate living cells [11]. This makes NPs suitable as both therapeutic agents and detection tools in many diseases, including cancer and infectious diseases [7,12,13]. The large surface areas of NPs also facilitate the modification of their surface by conjugation or loading with target molecules for sensing or delivery in therapeutic applications [14,15,16]. Multiple synthetic methods exist to generate NPs, including physical, chemical, and biological routes [17]. The physicochemical approaches have been used to create NPs of various shapes and sizes, with important agricultural, industrial, and medical applications [18,19]. However, these physicochemical methods use toxic chemicals for capping and reduction during the fabrication of NPs, which threaten the environment. Moreover, these toxic materials remain conjugated to the surfaces of the synthesized NPs, which reduces their biosafety to normal living cells [18,20]. Green synthesis methods have emerged to overcome these limitations.

In green synthesis approaches, the synthesis process mimics phenomena that occur in nature. Many living organisms, including bacteria [21], fungi [22], plants [23], and cyanobacteria [24], are able to convert bulk materials in their environment into nanoscale materials. Thus, in the laboratory, to prepare NPs via biological synthesis, the bulk material of interest (as a salt) is reduced using natural sources of reducing and stabilizing agents (macro- or microorganisms, or biomolecules, such as vitamins, proteins, and enzymes) [25]. This process is easy to perform and it requires no toxic material, as well as having the advantages of low cost and low energy consumption, and it is ecofriendly [18]. Several studies have reported that biogenic NPs have low toxicity against normal cells [26,27]. El-Naggar et al. [28] showed that SNPs synthesized using phycobiliprotein that was extracted from *Nostoc* sp. had fewer cytotoxic effects against normal WI38 and WISH cells as compared with 5-fuorouracil (a standard anticancer drug).

Silver NPs (SNPs) have unique physicochemical and biological features that make them attractive in various domains, including medicine, agriculture, and industry [18]. These particles are also a promising alternative therapeutic agents against many diseases, including microbial and viral infections [12,29,30] and cancer [24], and for the treatment of burns [31]. For example, an SNP-based bandage approved by the Food and Drug Administration as a treatment for burns is already commercially available under the trade name of Acticoat [31]. Moreover, several studies have reported the antiproliferative activity of SNPs toward different types of cancer cells, including MCF-7, HepG2, Caco-2, and HCT-116 cells [32,33]. 

The detailed mechanism by which NPs kill cancer cells is still under investigation. However, several studies attributed the bioactivity of NPs against cancer cells to two general strategies: (i) direct influence, in which the NPs interact directly with cellular components (proteins, DNA, antioxidants, enzymes, etc.) and cellular structures (such as cell membranes and organelles), leading to enhanced cell death [34] and (ii) indirect influence, in which the NPs induce the production of reactive oxygen species (ROS). This results in intensive oxidative stress, which causes DNA damage and protein modification and degradation, as well as imbalance in enzyme activities causing metabolic toxicity and cellular dysfunctions that finally lead to cell death [35,36]. Gopinath et al. [37] showed that SNPs disrupted normal cellular functions and membrane integrity, enhancing various apoptotic signaling genes of living cells and, thus, causing apoptosis. Satapathy et al. [36] reported that SNPs are *bona fide* anticancer drugs that act in a p53-dependent manner. Sanpui et al. [9] showed that a chitosan nanocarrier-based delivery of SNPs induced apoptosis in HT-29 cells by increasing production of ROS and stimulating caspase signaling pathways. This ability of SNPs to enhance apoptosis has the potential to be exploited in nanomedicine, as programmed cell death is key to overcoming diseases, including cancer [9,38]. 

Here, we have systematically screened for the first time the anticancer potential of green novel SNPs synthesized by *Nostoc* Bahar M (N-SNPs) against three different cancer cell lines (MCF-7, HepG2, and HCT-116) and further explored the underlying mechanisms. The cytotoxicity of N-SNPs against cancer cells was investigated by cell viability assays and morphological analyses using inverted light and electron microscopes. Analyses of antioxidants (glutathione (GSH) and glutathione peroxidase (GPx)) and enzymes (lactate dehydrogenase (LDH) and adenosine triphosphatase (ATPase)) were also performed. To investigate the multiplex origins of N-SNP-mediated apoptosis, the protein expression levels of anti-apoptotic markers (Akt, p-Akt, mTOR, and Bcl-2) and apoptotic markers (p53 and caspase 3) were determined. The results suggest that the N-SNPs are potential ecofriendly *bona fide* anticancer drugs and provide insight into the molecular mechanism of the cytotoxicity of *Nostoc*-mediated SNPs against cancer cells. 

## 2. Material and Methods

### 2.1. Materials

The MCF-7, HCT-116, and HepG2 cells were procured from the Medical Research Institute, Alexandria, Egypt. All cell culture reagents, media, and standard chemicals were procured from Sigma–Aldrich (St. Louis, MO, USA); the LDH assay kit (Colorimetric) and all antibodies (Table 1) were from Abcam (Cambridge, UK); and, the PiBind resin was from Expedeon (San Diego, CA, USA). 

### 2.2. Methods

#### 2.2.1. Preparation of N-SNP Suspension 

The methods used for extracellular synthesis of SNPs using *Nostoc* Bahar M sp. and for their physicochemical characterization are described in a previous publication [24]. In brief, *Nostoc* sp. culture was harvested and washed by centrifugation at 4000 rpm and the biomass pellets collected. Afterward, the pellets were freeze-dried and then crushed into fine powder utilizing a mortar and pestle. 20 mg of cyanobacterial powder was mixed with 20 mL of distilled water to boil at 30 °C for 24 h. The mixture was filtrated while using Whatman filter paper no.1 (Camlab, Cambridge, UK) and 10 mL of filtrate mixed with 90 mL of 1 mM of silver nitrate aqueous solution at ambient temperature for 24 h in the dark. After 24 h, the pellets were harvested and then washed at least three time using centrifugation at 10000 rpm. Afterward, the resultant pellets were spread on plates to dry at 40 °C for 24 h.

The SNPs appear as a fine black powder, with the particles having spherical shape and sizes that range between 8.5 and 26.44 nm (nanosize), with a mean size of 14.9 ± 0.56 nm. A stock solution of N-SNPs (1 mg/mL) was prepared in aspect complete Dulbecco’s modified Eagle’s medium (DMEM) and diluted to the required concentrations with cell culture medium. The N-SNP solution was filtrated using a 0.22-µm sterile microfilter before being injected into cancer cells.

#### 2.2.2. Cell Culture

MCF-7 breast cancer cells, HCT-116 colorectal adenocarcinoma cells, and HepG2 liver cancer cells were cultured following the standard protocols for established cell lines [32,33]. The cells were propagated in DMEM that was enriched with 10% fetal bovine serum and 50 U/mL penicillin/streptomycin, and then incubated in a humidified atmosphere containing 5% CO_2_ at 37 °C. The confluent cells were passaged using trypsin-EDTA.

#### 2.2.3. MTT Assay

The MCF-7, HCT-116, and HepG2 cells (1 × 10^4^ cells/well) were plated in 96-well plates in complete DMEM and they reached optimal population densities within 48 h. The cells were then exposed to increasing concentrations of the filtrated N-SNPs (31.25–1000 µg/mL) and incubated for 24 h in a humidified atmosphere containing 5% CO_2_ at 37 °C. After 24 h, the old medium was discarded and replaced with 100 µL fresh DMEM; then, 10 µL of 12 mM MTT stock solution was added to each well and maintained for 24 h. As a negative control, 100 µL of medium alone was mixed with 10 µL MTT solution under the same conditions. Subsequently, 100 µL/well of dimethyl sulfoxide (DMSO) was added to dissolve the formazan crystals [39]. The absorbance of the samples was recorded at 570 nm with a Benchmark Microplate ELISA reader (Bio-Rad Laboratories, Hercules, CA, USA). The cell viability percentage was evaluated while using the following formula: (*A*_Treated_ − *A*_Blank_)/(*A*_Control_ − *A*_Blank_) × 100.

The concentrations are necessary for 50% and 25% of growth inhibition (IC_50_ and IC_25_) were calculated using a sigmoidal curve of % cell viability versus logarithmic concentration of SNPs using Graph Pad Prism software [40].

#### 2.2.4. Morphological Change Estimation

Equal numbers of MCF-7, HCT-116, and HepG2 cells (1 × 10^4^ cells/ well) were seeded onto 12-well plates until sub-confluent and then treated with the corresponding IC_50_ and IC_25_ of biogenic N-SNPs (Table 2) and kept for 24 h at 37 °C. The untreated cells were cultured similarly as a negative control. After 24 h, morphological changes were detected while using an inverted light microscope (Optika, Ponteranica BG, Via Rigla, Italy).

#### 2.2.5. Transmission Electron Microscopy (TEM)

Ultrastructural alterations in the cells after treatment with N-SNPs were detected by TEM at 80 kV (Jeol 100 CX, Tokyo, Japan). In brief, HepG2, HCT-116, and MCF-7 cells (1 × 10^4^ cells/well) were seeded in a 25-cm^2^ cell culture flask until sub-confluent, exposed to N-SNPs at IC_50_ and IC_25_ (Table 2), and then incubated for 24 h at 37 °C. The cells were cultured similarly, but without treatment as a negative control. The cells were washed several times with phosphate-buffered saline (PBS) to remove excess unbound N-SNPs, trypsinized, and then washed again 4–5 times with PBS. The cell pellets were collected after centrifugation at 3000 rpm at 4 °C. The cells were immediately immersed in ice-cold 2.5% glutaraldehyde for 2 h at 4 °C, and then rinsed several times with 0.1 M PBS before being post-fixed with 1% osmium tetroxide (OsO_4_) for 2 h at 4 °C. Afterward, the specimens were serially dehydrated with graded ethanol (50%, 70%, 90%, and 100%), infiltrated with propylene oxide, and then embedded in Araldite-Epon mixture. The samples were cut into ultrathin (70 nm) sections with a glass knife on an LKB ultramicrotome and then double-stained on 200-mesh copper grids with 2% uranyl acetate and lead citrate for examination [41].

#### 2.2.6. Morphometric Measurements

The dimensions of cells, nuclei, and oval mitochondria (length and width) and of spherical mitochondria (diameter) of the three tested cell types were measured based on the TEM micrographs while using the ImageJ software (averaged over 10 cells).

#### 2.2.7. Membrane Integrity 

LDH assays were used to determine the effects of N-SNPs on the cell membrane integrity of MCF-7, HCT-116, and HepG2 cells, following the manufacturer’s instructions. In this assay, the toxicity of the N-SNPs can be determined by measuring the LDH level in the supernatant, which increases due to the cytosolic LDH enzyme leaking out of the cells via disrupted cellular membranes. Briefly, the three cell lines before and after treatment with N-SNPs at IC_25_ and IC_50_ (Table 2) were maintained at 37 °C for 24 h. Subsequently, in a new 96-well plate, 100 µL/well of each cell-free supernatant was mixed in triplicate with 100 µL of the LDH assay reaction mixture. The absorbance of the color produced after 3 h of incubation was estimated at a wavelength of 490 nm while using a plate reader [42]. This experiment was performed at least three times under consistent conditions.

#### 2.2.8. Estimation of ATPase Activity

ATPase activity before and after exposure of MCF-7, HepG2, and HCT-116 cells to different concentrations of N-SNPs was evaluated using a colorimetric ATPase assay kit, according to the method that was described by Andrés et al. [43] The supernatants of cells (10 µL) were mixed with 10 μL of PiBind resin to remove free inorganic phosphate (Pi). The amount of liberated Pi was evaluated by spectrophotometry (UV 2505, La Cienega Blvd., Los Angeles, CA 90034 U.S.A) at A_650_. For all experiments, calibration was performed while using a standard range of Pi concentrations. The experiments were performed at least three times under consistent conditions.

#### 2.2.9. Evaluation of Antioxidative Marker Levels

In brief, the tested cells were incubated with different concentrations of N-SNPs (IC_50_ and IC_25_) in a 25-cm^2^ cell culture flask for 24 h at 37 °C. Subsequently, the cells were scraped and washed twice with PBS before being centrifuged at 4 °C and 1500 rpm for 6 min. The pellets were collected and then sonicated at 15 W for 10 s (three cycles) to obtain the cell lysate. The GPx and GSH concentrations of the supernatant were evaluated.

For evaluation of GPx levels, 0.9 mL of the sample was mixed with the reaction mixture (50 mM potassium phosphate buffer (pH 7.0), 1 mM EDTA, 1 mM sodium azide, 0.2 mM NADPH, 1 U glutathione reductase, and 1 mM reduced glutathione) and maintained at 25 °C for 5 min. The reaction was started by the addition of 0.1 mL 2.5 mM hydrogen peroxide (H_2_O_2_), and the absorbance was estimated at 340 nm. Values were expressed as *n*mol of NADPH oxidized to NADP using an extinction coefficient of 6.2 × 10^3^ M^−1^ cm^−1^ at 340 nm. The activity of GPx was expressed in terms of *n* mol NADPH consumed/min/mg protein [44]. 

For the evaluation of GSH levels, the supernatant was mixed with a solution of cold (4 °C) 320 mM sulfosalicylic acid, 28 mM L-ascorbic acid, and 4 mM EDTA for protein precipitation. Subsequently, the mixture was centrifuged at 27,000 × 1q *g* (Hettich Zentrifugen MIKRO 200, Föhrenstraße, Tuttlingen, Germany) for 15 min, at 4 °C to remove precipitated proteins, and the GSH concentration was measured in the clear supernatant by the colorimetric method that was described by Beutler et al. [45]. Subsequently, 0.1 mL of supernatant was mixed with the reaction mixture (5,5′-dithiobis (2-nitrobenzoic acid) dissolved in 25 mM PBS, pH 7.0). The absorbance was measured at 412 nm. The GSH content was expressed as μM GSH per milligram. These experiments were performed at least three times under consistent conditions.

#### 2.2.10. Western Blotting Analysis

The expression levels of apoptotic and antiapoptotic proteins (tumor suppressor p53, caspase 3, protein kinase B (Akt), phosphorylated-Akt (p-Akt), mammalian target of rapamycin (mTOR) and B-cell lymphoma 2 (Bcl-2)) were measured by western blotting. MCF-7, HCT-116, and HepG2 cells (1 × 10^4^) were separately cultured in six-well plates until they reached approximately 90% confluence. The cells were treated with N-SNPs at IC_25_ and IC_50_ and then incubated for 24 h at 37 °C. Afterwards, the media were discarded, and the cells were resuspended three times with ice-cold PBS, detached, and then centrifuged at 1500 rpm at ambient temperature. The cells were homogenized with protein lysis buffer (50 mM Tris pH 7.5, 150 mM NaCl, 1% Triton X-100, 0.1% sodium dodecyl sulfate (SDS)) in the presence of protease inhibitor. The concentration of protein was detected by the Bradford method [46]; 30 µg of the total protein sample was loaded into a mini-gel well while using a special gel loading tip, which was submerged in a migration buffer containing SDS. To visualize the fixed proteins, the gel was stained with 0.1% Coomassie blue R- 250 for 2 h, and then destained with a solution of glacial acetic acid, followed by methanol and water. After SDS polyacrylamide gel electrophoresis, the proteins were transferred to a *Hybond*^™^ nylon membrane (GE Healthcare) using a TE62 standard transfer tank with cooling chamber (Hoefer Inc. Holliston, Massachusetts, United States) and then detected using anti-Akt, anti-p-Akt, anti-mTOR, anti-Bcl-2, anti-p53, and anti-caspase 3. β-actin was used as a housekeeping protein. A gel documentation system (Geldoc-it UVP, Loughborough, Leicestershire, England) was used for the data analysis with Total Lab analysis software (www.totallab.com), version 1.0.1. 

#### 2.2.11. Statistical Analysis

All the data from three independent replicates are presented as mean ± SEM. Statistical analyses were performed using one-way analysis of variance with the Prism 8.3 software (GraphPad Software Inc., La Jolla, CA, USA). The results were considered to be statistically significant at *p* < 0.01, *p* < 0.001, *p* < 0.0003, and *p* < 0.0001. The ImageJ software (National Institutes of Health, Bethesda, MD, USA) was used for morphometric analysis. 

## 3. Results

### 3.1. Cytotoxic Activity of N-SNPs Against MCF-7, HCT-116, and HepG2 Cells

**Figure 1:**
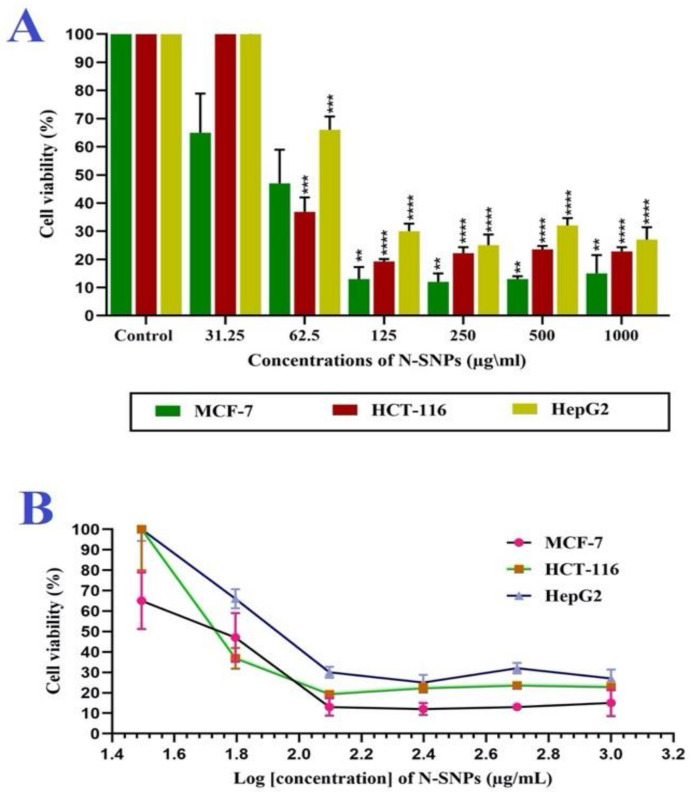
Antiproliferative activity of *Nostoc*-mediated synthesized silver nanoparticles (N-SNPs). (**A**) Percentages of viable breast, liver and colon cancer (MCF-7, HCT-116, and HepG2, respectively) cells before and after treatment with different concentrations of N-SNPs for 24 h. (**B**) Cell viability percentages and log concentration of N-SNPs. All of the data were collected from independent experiments performed in triplicate and are presented as mean ± standard error of the mean (SEM). *p*-values were calculated versus untreated cells: **** *p* < 0.0001, *** *p* < 0.0003, **p < 0.001.

The toxicity of green N-SNPs (31.25–1000 µg/mL) against the three cancer cell lines was evaluated while using cell viability assays. The results showed that N-SNPs significantly inhibited the proliferation of all tested cells in a dose-dependent manner as compared with untreated cells (Figure 1A). The cytotoxic potency of N-SNPs against the cells was variable; the MCF-7 cells were the most sensitive to N-SNPs, followed by HCT-116 and HepG2 cells, with IC_50_ values of 54, 56, and 80 µg/mL, respectively (Figure 1B and Table 2).

### 3.2. Morphological Changes of Cells Treated with N-SNPs

**Figure 2:**
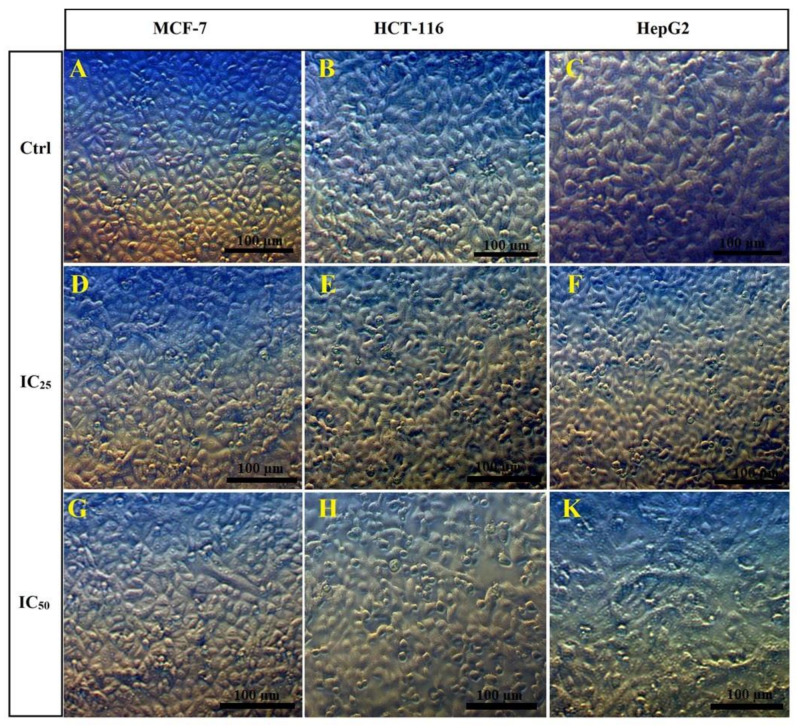
Morphological features before and after treatment of MCF-7, HCT-116, and HepG2 cells with N-SNPs at IC25 and IC50 for 24 h. (**A**–**C**) Untreated MCF-7, HCT-116, and HepG2 cells, respectively. (**D**–**F**) MCF-7, HCT-116, and HepG2 cells treated with N-SNPs at IC25, respectively. (**G**–**K**) MCF-7, HCT-116, and HepG2 cells treated with N-SNPs at IC50, respectively. Scale bar = 100 µm.

The morphological changes in MCF-7, HCT-116, and HepG2 cells after being treated with different concentrations of N-SNPs (IC_25_ and IC_50_) were examined by inverted light microscopy. All of the selected untreated cells showed normal morphological appearance and distribution, and formed a confluent monolayer of cells attached to the plate with almost 90% confluence and a low distance between cells (Figure 2A–C). By contrast, all of the tested cells showed detectable morphological alterations after treatment with both concentrations of N-SNPs, in a dose-dependent manner; these alterations included cell detachment, shrinkage, and rounding, a restricted spreading pattern, and clustering (Figure 2D–K). However, cells that were treated with N-SNPs at IC_50_ exhibited more drastic changes as compared with cells treated at IC_25_; there was a greater decrease in intracellular connections between the cells, a larger number of floating dead cells, and more lysed cells with incomplete nucleus and cytoplasm, the cell volume was decreased, and more rounded cells were generated (Figure 2G–K).

### 3.3. Membrane Integrity 

**Figure 3:**
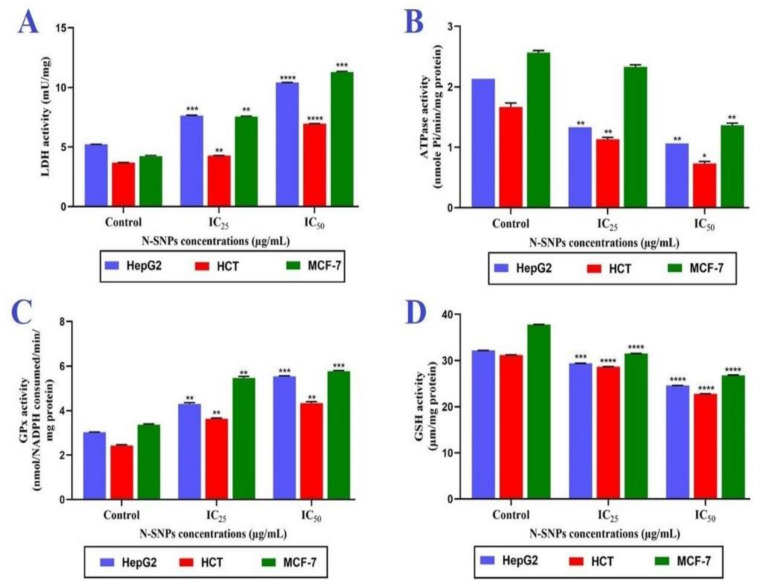
Influence of N-SNPs at IC25 and IC50 on enzymes and antioxidants in MCF-7, HCT-116, and HepG2 cells. (**A**) Lactate dehydrogenase (LDH) enzyme, (**B**) adenosine triphosphatase (ATPase), (**C**) glutathione peroxidase (GPx), and (**D**) glutathione (GSH). All of the data were collected from independent experiments performed in triplicate and are presented as mean ± SEM. *p*-values were calculated versus untreated cells: **** *p* < 0.0001, *** *p* < 0.0003, **p < 0.001, *p < 0.01.

In all tested cell lines, the LDH levels were significantly increased in a dose-dependent manner 24 h after treatment with N-SNPs at both IC_25_ and IC_50_ (Figure 3A). However, IC_50_ was the most potent concentration, causing a significant increase in LDH levels in all tested cells as compared with those that were treated at IC_25_ and untreated cells. Among the treated cells, the MCF-7 cells showed the greatest increase in LDH levels, followed by HepG2 and HCT-116, after exposure to both concentrations of N-SNPs. 

### 3.4. ATPase Assay

The metabolic toxicity of N-SNPs against the three tested cell lines was determined by measuring ATPase activity before and after treatment with both concentrations of N-SNPs. In all tested cancer cells (MCF-7, HCT-116, and HepG2), the ATPase levels significantly decreased in a dose-dependent manner after exposure to N-SNPs at IC_25_ or IC_50_ (Figure 3B). Of the three cell lines, the MCF-7 cells showed the greatest decrease in ATPase levels.

### 3.5. Antioxidative Markers 

The oxidative stress that was caused by N-SNPs was evaluated by measuring the activities of antioxidants (GPx and GSH). The results showed that the GPx levels significantly increased and GSH levels significantly decreased in a dose-dependent manner in all three cell types treated with both concentrations of N-SNPs (IC_25_ and IC_50_) (Figure 3C,D). The MCF-7 cells treated with N-SNPs showed the greatest imbalance in GPx and GSH levels.

### 3.6. Ultrastructural Changes of Treated Cells with N-SNPs

**Figure 4:**
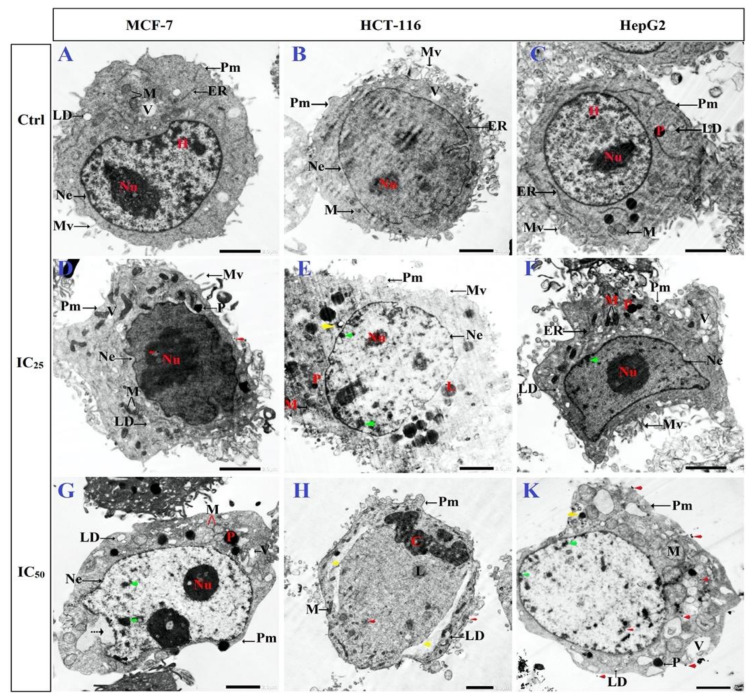
Transmission Electron Microscopy (TEM) micrographs of MCF-7, HCT-116, and HepG2 illustrating ultrastructural features before treatment (**A**–**C**), after exposure to N-SNPs at IC25 (27, 28, and 40 µg/mL, respectively) (**D**–**F**), and after exposure to N-SNPs at IC50 (54, 56, and 80 µg/mL, respectively) (**G**–**K**). Images show irregular plasma membrane (Pm), low distribution or absence of microvilli (Mv), and endoplasmic reticulum (ER), high incidence of lipid droplets (LD), mitochondria (M), lysosomes (L), vacuoles (V), large vacuoles (headed yellow arrow), autophagic vacuoles (yellow arrow), cytoplasmic dissolution (cut arrow), irregular nuclear envelope (Ne), marginated and aggregated heterochromatin (green arrow), and condensed chromatin (C). Red arrows show the distribution of N-SNPs in cytoplasm, plasma membrane, and organelles. Scale bar = 2 µm.

TEM was used to investigate the toxic effects of N-SNPs on cellular structures and their interactions with organelles. The TEM micrographs of untreated MCF-7, HCT-116, and HepG2 cells showed typical morphological features of tumor cells, including intact cellular membranes; numerous microvilli and membrane blebbing on the surface of plasma membranes; centric large nuclei with obvious nucleoli, surrounded by intact nuclear membranes; and, a normal distribution of heterochromatin. In addition, there was a typical distribution of cellular organelles in the cytoplasm, including electron-dense pleomorphic mitochondria, lipid droplets (LDs), few cytoplasmic vacuoles, dense secretory granules that may have been peroxisomes, and endoplasmic reticulum (ER) (Figure 4A–C).

**Figure 5:**
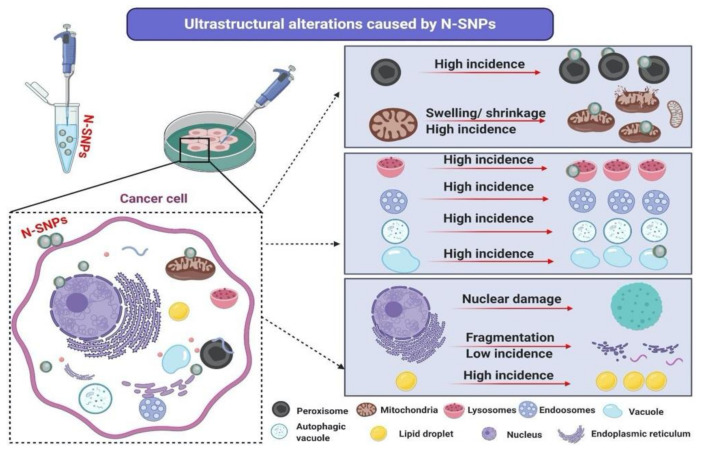
Ultrastructural alterations in cancer cells caused by N-SNPs.

TEM micrographs of MCF-7, HCT-116, and HepG2 cells that were exposed to N-SNPs at concentrations of 27, 28, and 40 µg/mL, respectively, exhibited several morphological changes indicating cell injury and inactivation (unsteady metabolic state). These subcellular changes included irregular cell membranes and a reduction in microvilli and membrane blebbing on the cell surface, as well as cytoplasm condensation (Figure 4D–F). Mitochondria were peripherally aggregated in the cytoplasm, and fragmented oval mitochondria were observed. In addition, oval and spherical mitochondria became markedly swollen in MCF-7 and HCT-116 cells that were treated with N-SNPs, and showed shrinkage in HepG2 cells treated with N-SNPs. In addition, the numbers of cellular organelles showed changes when compared with the corresponding control cells (Figure 5). 

**Figure 6:**
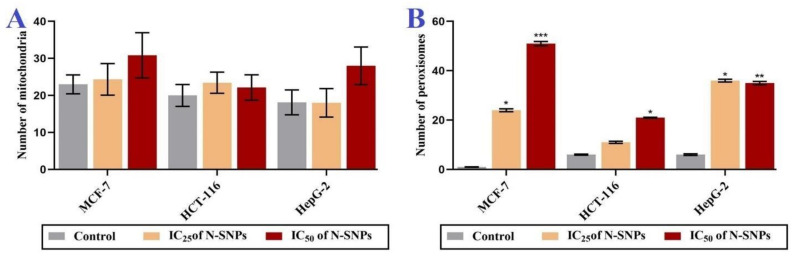
Changes in distribution of (**A**) number of mitochondria and (**B**) number of peroxisomes in MCF-7, HCT- 116, and HepG2 cells before and after treatment with N-SNPs at IC25 and IC50 for 24 h. Data represent averages of measurements from 10 cells and are presented as mean ± SEM. *p*-values were calculated versus untreated cells: *** *p* < 0.0001, ** *p* < 0.001, * *p* < 0.01.

**Figure 7:**
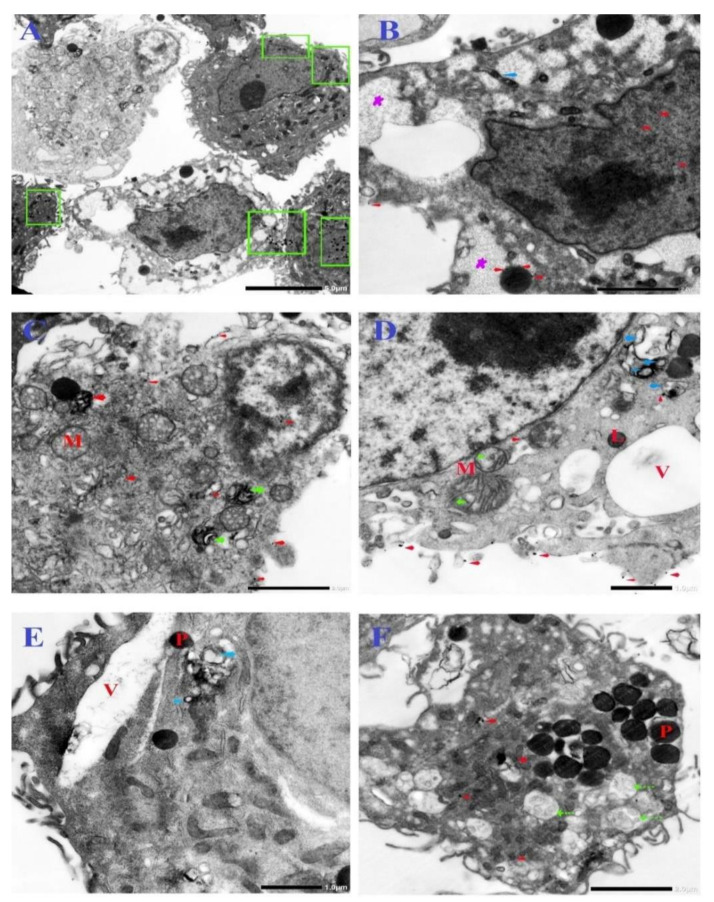
TEM micrographs of MCF-7 (**A–E**) and HepG2 (**F**) cells treated with N-SNPs at IC25. (**A**) Distribution of dark electron-dense granules that may indicate N-SNPs in cytoplasm (green square). (**B**) Cytoplasm dissolution (violet star), swollen oval mitochondria (blue arrow), scattering of N-SNPs in nuclear matrix and peroxisomes (red arrow). (**C**) Scattering of autophagic vacuoles (green arrow), swelling of spherical mitochondria (M), and distribution of N-SNPs in cytoplasm and plasma membrane (red arrow). (**D**–**E**) Autophagic vacuoles (blue arrow), mitochondrial damage (green arrow), and attachment of NPs to cell membrane, mitochondria, and autophagic vacuoles (**E**). (**F**) Extensive appearance of early endosomes (green cut arrow) and peroxisomes (P), as well as scattering of N-SNPs in cytoplasm (red arrow). V, vacuole; P, peroxisome; L, lysosome; M, mitochondria. Scale bar = 5 µm (**A**), 2 µm (**B, C,** and **F**), and 1 µm (**D** and **E**).

For instance, the numbers of mitochondria and peroxisomes in cells that were treated with N-SNPs increased in comparison with those in untreated cells (Figure 6). The amount of ER in the cytoplasm decreased, and, the number of LDs increased; some of the LDs appeared to be fused with oval mitochondria (Figure 4). Moreover, many autophagic vacuoles and less dense endosomes were distributed in the cytoplasm (Figure 7). Folded nuclear membrane and marginated chromatin were also observed. Of note, small, dark electron-dense granules were scattered in the cytoplasm, some of which were attached to the surfaces of peroxisomes, mitochondria, nuclear and plasma membranes, and microvilli and membrane blebs (Figure 7); these particles are believed to have been the SNPs. 

**Figure 8:**
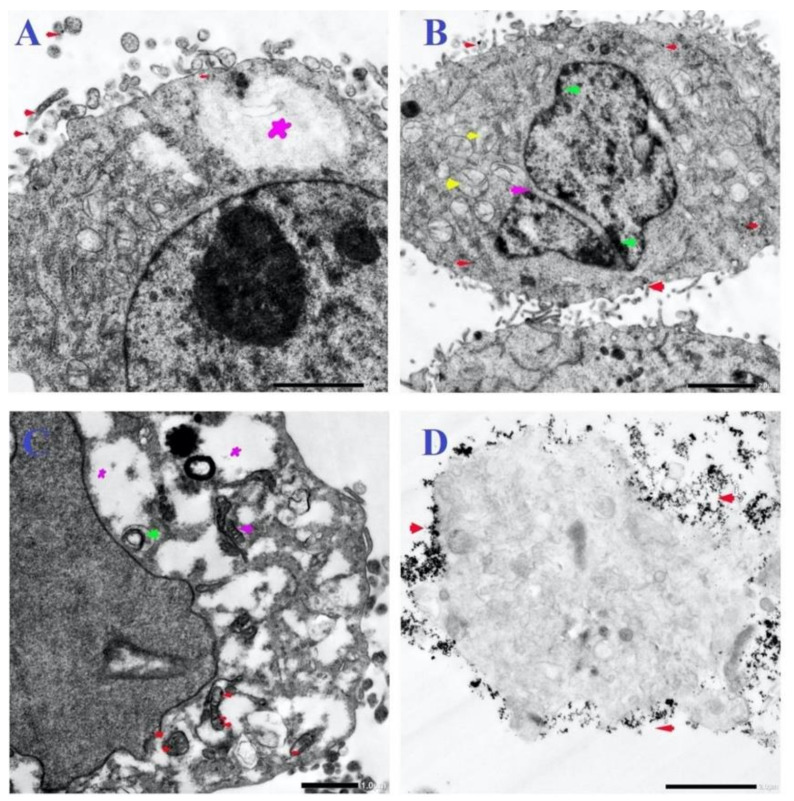
TEM micrographs of HCT-116 (**A-B**), MCF-7 (**C**), and HepG2 (**D**) cells treated with N-SNPs at IC50. (**A**) Cytoplasm dissolution (violet star), N-SNPs attached to membrane blebbing and microvilli and scattered in cytoplasm (red arrow). (**B**) Swollen mitochondria with damaged cristae (yellow arrow), fragmented nucleus (purple arrow) with margined chromatin (green arrow), N-SNPs attached to membrane blebs and scattered in cytoplasm (red arrow). (**C**) Cytoplasmic dissolution (violet star), autophagic vacuoles (green arrow), fragmented oval mitochondria (violet arrow), attachment of N-SNPs to mitochondria (red arrow). (**D**) Apoptotic body and attachment of NPs to plasma membrane (red arrow). Scale bar = 2 µm (**A, B** and **D**) and 1 µm (**C**).

Similarly, micrographs of MCF-7, HCT-116, and HepG2 cells that were treated with N-SNPs at concentrations of 54, 56, and 80 µg/mL, respectively, showed distinct features of cellular apoptosis, including the disintegration of cell membranes, disappearance of microvilli and membrane blebbing, and cytoplasmic condensation and dissolution (Figure 4G–K). The mitochondria were found to be aggregated near the cytoplasmic membrane or nuclear envelope. The sizes of spherical and oval mitochondria increased after treatment with N-SNPs in MCF-7 and HCT-116 cells, but decreased in HepG2 cells. The numbers of mitochondria and peroxisomes distributed within the cytoplasm were higher in treated cells compared with untreated cells (Figure 6). A large number of primary lysosomes, condensation of lipid droplets, extensive cytoplasmic vacuoles, and a sparse distribution of ER were observed. Irregular or absent nuclear envelopes were only observed in MCF-7 and HCT-116 cells that were treated with N-SNPs at IC_50_. The nuclei of MCF-7 and HepG2 cells possessed peripheral heterochromatin restricted to the nuclear membrane, and aggregated patches or islands of heterochromatin were scattered within the nuclear matrix; however, the heterochromatin of HCT-116 cell nuclei was peripherally agglomerated near the cellular membrane. Moreover, some HCT-116 cells treated with N-SNPs at IC_50_ had fragmented nuclei (Figure 8). Dark, electron-dense spherical particles were attached to cellular and nuclear membranes and to the surfaces of mitochondria, vacuoles, and lipid bodies; these particles may have been SNPs (Figure 8).

**Figure 9:**
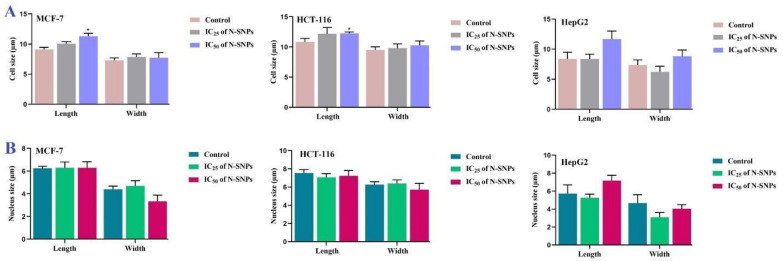
Morphometric analysis (µm) of (**A**) cell size (length and width) and (**B**) nucleus size (length and width) of MCF-7, HCT-116, and HepG2 cells before and after treatment with N-SNPs at IC25 and IC50. Data represent averages of measurements of 10 cells and they are presented as mean ± SEM. *p*-values were calculated versus untreated cells: * *p* < 0.01.

The analysis of morphometric measurements revealed that both concentrations (IC_25_ and IC_50_) of N-SNPs induced changes in cell, nuclear, and mitochondrial dimensions as compared with control cells. Treatment with N-SNPs at IC_25_ led to non-significant increases in cellular lengths and widths in MCF-7 and HCT-116; however, there was no change in HepG2 cell length and a non-significant decrease in their width. Treatment with N-SNPs at IC_50_ resulted in significant increases in lengths of MCF-7 and HCT-116 cells and an insignificant increase in length of HepG2 cells, as well as non-significant increases in cellular widths of all three cell types (Figure 9A). 

Similarly, N-SNPs at IC_25_ led to non-significant increases in both nuclear dimensions of MCF-7 cells, as well as a non-significant decrease in HCT-116 nuclear length and a non-significant increase in width. The nuclei of HepG2 cells treated with N-SNPs at IC_25_ showed non-significant decreases in both length and width. N-SNPs at IC_50_ resulted in non-significant increases in nuclear lengths in MCF-7, HCT-116, and HepG2 cells, and insignificant decreases in nuclear widths (Figure 9B).

**Figure 10:**
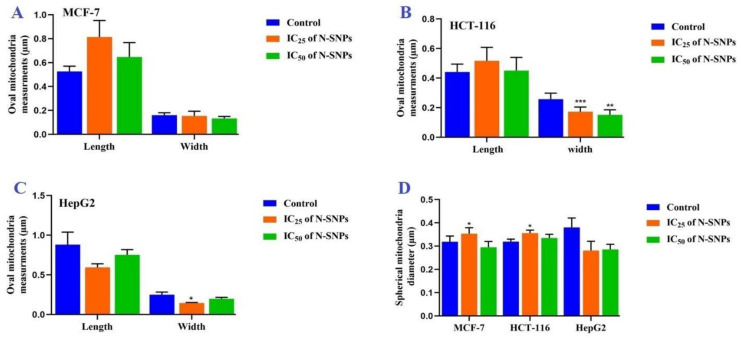
Morphometric analysis (µm) of (**A-C**) oval mitochondria dimensions and (**D**) spherical mitochondria diameters in MCF-7, HCT-116, and HepG2 cells before and after treatment with IC25 and IC50 of N-SNPs. Data represent averages of measurements from 10 cells and are presented as mean ± SEM. *p*-values were calculated versus untreated cells: *p < 0.01.

In addition, both concentrations of N-SNPs led to significant changes in the dimensions of oval and spherical mitochondria (Figure 10A–C). In MCF-7 and HCT-116 cells, exposure to both concentrations of N-SNPs caused changes in the dimensions of oval mitochondria, with non-significant increases in length and significant decreases in width. Both concentrations of N-SNPs resulted in decreased oval mitochondria dimensions in HepG2. 

In MCF-7 cells that were treated with N-SNPs at IC_25_, there was a significant increase in spherical mitochondria diameter (SMD), whereas treatment with N-SNPs at IC_50_ resulted in a non-significant decrease in SMD. By contrast, both concentrations of N-SNPs resulted in a significant increase in SMD in HCT-116 cells, but a non-significant decrease in HepG2 cells (Figure 10D). Interestingly, greater changes in oval mitochondria dimensions and SMD occurred in cells that were treated with N-SNPs at IC_25_ as compared with IC_50_. 

### 3.7. Western Blotting Technique

**Figure 11:**
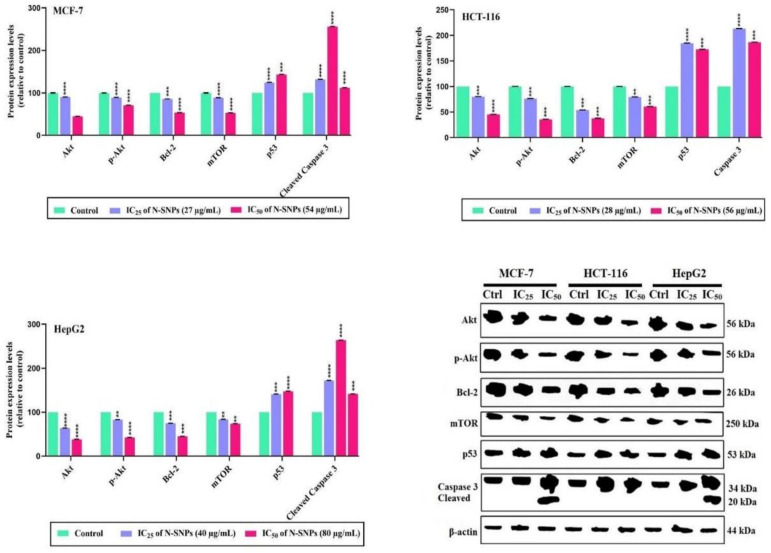
Effects of N-SNPs (IC_25_ and IC_50_) on antiapoptotic (protein kinase B (Akt), phosphorylated-Akt (p-Akt), B-cell lymphoma 2 (Bcl-2) and mammalian target of rapamycin (mTOR)) and apoptotic (tumor suppressor (p53) and caspase 3) protein expression levels in MCF-7, HCT-116, and HepG2 cells. All of the data were collected from independent experiments performed in triplicate and are presented as mean ± SEM. *p*-values were calculated versus untreated cells: **** *p* < 0.0001, *** *p* < 0.0003, **p < 0.001.

The ability of N-SNPs (at IC_25_ and IC_50_) to induce apoptotic cell death pathways in the three tested types cells (MCF-7, HCT-116, and HepG2) was evaluated against several apoptotic marker proteins (Akt, p-Akt, Bcl-2, mTOR, p53, and caspase 3) using western blotting (Figure 11, the whole WB images can be found in Figures S2–S8).

N-SNPs at IC_25_ and IC_50_ caused the downregulation of apoptosis-evading protein (Akt, p-Akt, Bcl-2, and mTOR) expression level in MCF-7, HCT-116, and HepG2 cells in a dose-dependent manner. N-SNPs at IC_50_ caused a greater decrease in Akt, p-Akt, Bcl-2, and mTOR protein expression levels when compared with N-SNPs at IC_25_ and no treatment. 

By contrast, protein expression levels of p53 and caspase 3 were increased after treatment of MCF-7, HCT-116, and HepG2 cells with N-SNPs at IC_25_ or IC_50_. Similarly, the higher concentration of N-SNPs (IC_50_) caused a greater increase in both p53 and caspase 3 protein expression levels. Only MCF-7 and HepG2 cells that were treated with N-SNPs at IC_50_ showed cleavage of caspase 3; their western blots exhibited two protein bands (34 and 20 kDa, respectively), whereas that of HCT-116 only showed one caspase 3 protein band (34 kDa). 

## 4. Discussion 

In this study, we screened, for the first time, the cytotoxic activity of SNPs that were synthesized using novel cyanobacteria strain *Nostoc* Bahar M against three types of cancer cells, MCF-7, HCT-116, and HepG2, and investigated the possible mechanism of action. The results showed that the N-SNPs were cytotoxic; they caused cellular and subcellular morphological alterations, enhanced oxidative stress and metabolic toxicity, and eventually induced apoptotic cell death via downregulating the expression of Akt, p-Akt, mTOR, and Bcl-2 proteins and upregulating the expression of p53 and caspase 3 proteins 

Cell viability assays showed that the N-SNPs acted as potent antiproliferative agents against MCF-7, HCT-116, and HepG2 cells (with IC_50_ values of 54, 56, and 80 µg/mL, respectively) in a dose-dependent manner. A recent study showed that SNPs (4.5–26 nm) synthesized using cyanobacteria *Desertifilum* sp. had dose-dependent cytotoxic activity against various cancer cell types, including HepG2, MCF-7, and Caco-2 cells (IC_50_ values of 32, 58, and 90 µg/mL, respectively). Similarly, Bin-Meferij and Hamida reported that SNPs (14.9 nm) that were synthesized using *Nostoc* Bahar M sp. significantly inhibited the growth of colon cancer cells with an IC_50_ of 150 µg/mL in a dose-dependent manner. 

Furthermore, SNPs produced by *Piper longum* fruit extract showed cytotoxic activity against MCF-7 cells with an IC_50_ of 67 µg/mL [47]. SNPs that were fabricated by *Lavandula dentata* leaf extracts (284.5 nm) exhibited stronger antiproliferative activity against HCT-116 (IC_50_ 59.79 µg/mL) than those that were synthesized by *Olea chrysophylla* extract (328.6 nm; IC_50_ 99.35 µg/mL) [48]. The remarkably high degree of cell death caused by N-SNPs could be due to their small size and strong affinity to cell membranes, which facilitates their diffusion into cells; in addition, the *Nostoc* biomaterial coat surrounding SNPs may increase their stability and aid in intracellular uptake. 

Inverted light micrographs showed that both concentrations of N-SNPs (IC_25_ and IC_50_) caused morphological changes; however, IC_50_ caused more drastic apoptosis-related alterations, including cell shrinkage, detachment, clustering, and rounding [24,49]. These cytomorphological changes may be attributed to the small size of N-SNPs, which enables them to interact with cellular structures, such as membranes, mitochondria, endoplasmic reticulum, and the nucleus, and cellular components, such as nucleic acids, proteins, and enzymes, leading to cellular dysfunction and cell death [24].

Analyses of enzymes and antioxidants showed that both concentrations of N-SNPs led to imbalances in ATPase, LDH, GPx, and GSH activities. The LDH leakage assay is used to estimate the toxicity of drugs against living cells based on the leakage of intracellular LDH into the extracellular matrix via impaired cellular membranes. LDH is a soluble cytoplasmic enzymes that exists in nearly all cells and it is liberated into the extracellular space through damaged plasma membranes [50,51]. The significant increase in LDH levels observed in the assay revealed that N-SNPs caused membrane disruption that influenced membrane integrity and permeability [52]. Yuan et al. [53] showed that SNPs (1 µM) alone or conjugated with camptothecin (1:1 µM) increased LDH leakage levels in HeLa cells, which indicated that SNPs influence plasma membrane integrity. 

The decrease in ATPase activity after exposure of cells to N-SNPs could be attributed to the induction of oxidative stress, which inhibits the activity and expression of ATPase, resulting in imbalances in cellular homeostasis and eventual damage to cells. It could also be due to the direct interaction of N-SNPs with ATPase or mitochondrial membranes, resulting in the denaturation of enzymes and mitochondrial dysfunction. Chen et al. [54] showed that ZnO NPs caused cytotoxicity in murine photoreceptor cells via potassium channel blocking and Na+/K+-ATPase suppression. Kandil et al. [55] showed that gallium NPs caused a decrease in ATPase levels in Ehrlich carcinoma-bearing mice and, when accompanied by low gamma radiation intensity, significantly increased ATPase enzyme activity. They also found that gallium influenced cell membrane permeability and ATPase activity. 

Antioxidants and antioxidant enzymes are the primary defense system against oxidative stress. In the first line of defense, antioxidant enzymes, such as superoxide dismutase and catalase, convert ROS into less damaging materials. The second tier of defense involves enzymes, such as GPx and glutathione S-transferases (GSTs), which detoxify the reactive intermediates. GSH plays an important part in the defense mechanism against oxidative stress as a free-radical scavenger and it can conjugate reactive intermediates via GSTs and GPx. Any imbalance in the activities of these antioxidants results in intensive oxidative stress [56,57]. The significant increase in GPx activity and decrease in GSH levels observed in the current study indicate that N-SNPs may stimulate the production of ROS, thereby inducing oxidative stress [53]. These data are in accordance with the findings of Petković et al. [58] who reported that TiO_2_ NPs caused a significant increase in GPx activity and decrease in GSH activity in HepG2 cells when compared with untreated cells. Al-Sheddi et al. [59] reported that the exposure of HeLa cells to different concentrations of SNPs (5, 10, and 25 μg/mL) caused increases in GSH levels of 40%, 55%, and 69%, respectively, as compared with untreated cells. 

The TEM images showed that both concentrations of N-SNPs induced several cellular alterations that were related to cell death in a dose-dependent manner (Figure 5). These ultrastructural alterations may have been caused by direct interactions between NPs and cell structures, and/or via the induction of oxidative stress by NPs, leading to cell damage [32,60,61].

The morphological changes that appeared after treatment of cells with N-SNPs can be divided into three general subcellular alteration patterns. The first pattern involves changes related to cell borders, including cell membrane disruption, folded membranes, and disappearance or reduced incidence of microvilli and membrane blebbing [62]. These changes were consistent with the results of the LDH enzyme analysis, which indicated that N-SNPs influenced membrane integrity and permeability. Moreover, the reduction in microvilli and plasma membrane blebbing after exposure to N-SNPs indicated that these NPs may influence the metastatic characteristics of cancer cells [63]. Sathishkumar et al. [64] showed that SNPs biosynthesized using an aqueous leaf extract of *Alternanthera tenella* significantly inhibited MCF-7 cell proliferation and migration. 

The second pattern comprised changes that occurred in the cytoplasm, including cytoplasmic dissolution and condensation, and those that occurred in cellular organelles. In MCF-7 and HCT-116 cells, N-SNPs increased the swelling of mitochondria and caused damage to their outer and inner membranes as well as their matrix and cristae. This suggests that the mechanism by which N-SNPs are toxic to cells involves their ability to induce oxidative stress via increasing ROS production; specifically, an increase in swelling of mitochondria enhances membrane fluidity and ROS generation and, thus, inhibits the respiration process [65]. These results were consistent with the data from the antioxidant analysis, which suggested that N-SNPs induced oxidative stress. On the other hand, HepG2 cells that were treated with N-SNPs showed a different response, in which the mitochondria size was decreased, which indicated that N-SNPs may exert their toxic effects against HepG2 cells by causing abnormalities in mitochondrial function [66]. It is clear that the response of cancer cells toward N-SNPs differs according to the cell type. 

An increase in number of mitochondria is associated with the use of stored cellular energy reserves in response to nutrient deprivation or exercise [67]. Thus, the intensive stress caused by N-SNPs may cause an increase in the number of mitochondria in cells, in order to generate more energy and avoid this stress [68]. Moreover, the dose-dependent increase in number of peroxisomes observed in all tested cells after exposure to N-SNPs may indicate that N-SNPs kill cancer cells via inducing oxidative stress, as ROS can be generated in peroxisomes by the production of H_2_O_2_ during fatty acid oxidation [69,70]. The high incidence of LDs observed in cells that were treated with N-SNPs indicates that N-SNPs may impair lipid catabolic activity, or that the use of fatty acids by cells may decrease, owing to nanotoxicity. 

Khatchadourian and Maysinger showed that the accumulation of LDs is a biomarker of oxidative stress and lipid homeostasis [71]. The fragmentation and dilation of ER and sparse distribution of ER observed in cells treated with N-SNPs indicate that these NPs caused ER stress and influenced the protein synthesis process [72]. These observations are consistent with the western blotting results, in which Akt and mTOR protein activity decreased after the treatment of cells with N-SNPs. In cancer cells, protein synthesis relies on mTOR signaling, in which the activation of mTOR activity enhances S6K and then S6 protein expression to synthesize proteins [73,74]. Thus, the downregulation of these proteins may impair protein synthesis. Kuang et al. [75] studied the effects of ZnO NPs on mouse liver and found that the NPs induced ER stress over an extended period of time, which activated apoptotic cell death pathways. The increases in numbers of lysosomes, endosomes, and autophagic vacuoles demonstrate that the N-SNPs have the potential to induce apoptosis as well as autophagic pathways [69,76]. 

The third pattern of subcellular alterations comprised nuclear alterations, such as disappearance or irregularities of the nuclear membrane, nucleoagglomeration, and dominant distribution of marginated heterochromatin and condensed chromatin. These observations indicated that N-SNPs may induce cell apoptosis by causing genetic damage [77]. This is consistent with data from the western blotting assay, in which p53 protein expression increased in all tested cells after exposure to N-SNPs, indicating DNA damage [36]. 

In addition, N-SNPs were successfully ingested into cancer cells and they were located in various cellular positions, including membranes, mitochondria, LDs, and peroxisomes; this may have been because their smaller size, charge, and bio-coat facilitated the internalization of the N-SNPs [68,78]. All of the previous observations indicate that N-SNPs induce cancer cells to undergo cell death via direct interactions with various organelles and biomolecules and/or via inducing oxidative stress, which causes organelle dysfunction and morphological changes leading to cell death. 

N-SNPs enhanced cell death pathways by suppressing the expression of antiapoptotic proteins (Akt, p-Akt, mTOR, Bcl-2) and promoting the activity of apoptotic proteins (p53 and caspase 3), possibly via inducing oxidative stress and/or through interactions with cellular components and organelles, such as DNA and mitochondria. The tumor suppressor p53 has a crucial role in the response to cellular stress; it regulates many genes that are involved in numerous cellular process, including DNA damage repair, cell cycle arrest, apoptosis, and cell survival [79]. The observed upregulation of p53 protein may indicate that N-SNPs directly and/or indirectly interact (via oxidative stress) with DNA, causing DNA damage that stimulates cell death pathways [36]. This would be in accordance with the TEM images, which showed that N-SNPs caused nuclear morphological alterations, nucleoagglomeration, and nuclear damage. 

Caspase 3 is a well-known major executor of the apoptosis pathway that orchestrates cellular destruction with proteolytic cascades [80]. Bcl-2 is a crucial protein that is involved in the mitochondrial apoptosis pathway, with the ability to control the permeability of the mitochondrial inner membrane [81,82]. Our results showed that N-SNPs increased the expression levels of caspase 3 and decreased the expression of Bcl-2, which suggests that N-SNPs may induce apoptosis via the mitochondrial signaling pathway [9,83]. 

**Figure 12:**
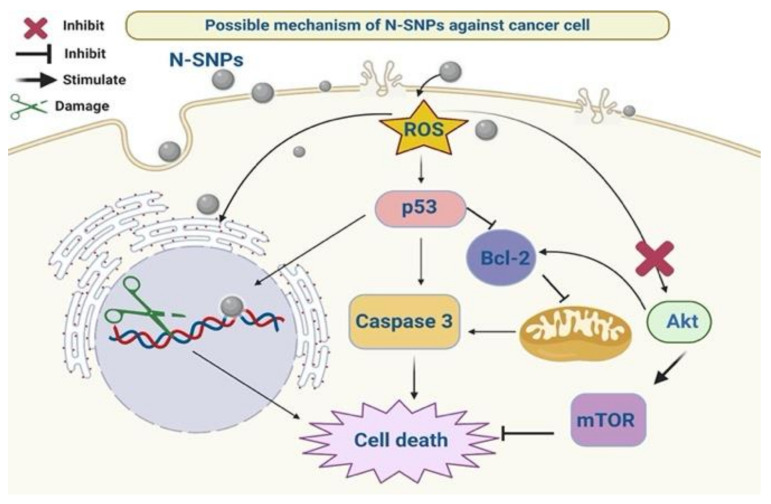
The possible molecular mechanism of N-SNPs against cancer cells.

Yao et al. [84] showed that PI3K/Akt/mTOR signaling is one of the classical pathways that suppresses apoptosis. Here, N-SNPs decreased the expression levels of Akt, p-Akt, and mTOR proteins, which suggests that N-SNPs suppress cell survival pathways via inhibiting mTOR signaling [36,83]. In addition, phosphorylated Akt can regulate cellular functions by regulating the phosphorylation of its downstream substrates, for example, controlling apoptosis via the regulation of Bcl-2 activity [85]. The observed imbalance in apoptosis-mediating proteins suggests that N-SNPs enhance programmed cell death by stimulating p53 and caspase 3 signaling, as well as suppressing cell survival pathways via inhibiting mTOR signaling [36,74]. Thus, we can hypothesize that N-SNPs have a broad range of cell-killing strategies, owing to their influence on various molecular pathways (Figure 12). 

The influence of N-SNPs on apoptosis-mediating proteins of MCF-7 and HepG2 cells was dose dependent; however, p53 and caspase 3 proteins in HCT-116 cells exhibited a different response, showing the highest expression levels after exposure to N-SNPs at IC_25_ rather than at IC_50_. This suggests that these apoptotic proteins are stabilized at low concentrations of NPs [86]. Thus, we suggest that the potential of NPs to induce cell death in cancer cells may be dependent on the concentration of NPs, the cell type, and cellular resistance.

## 5. Conclusions

**Figure 13:**
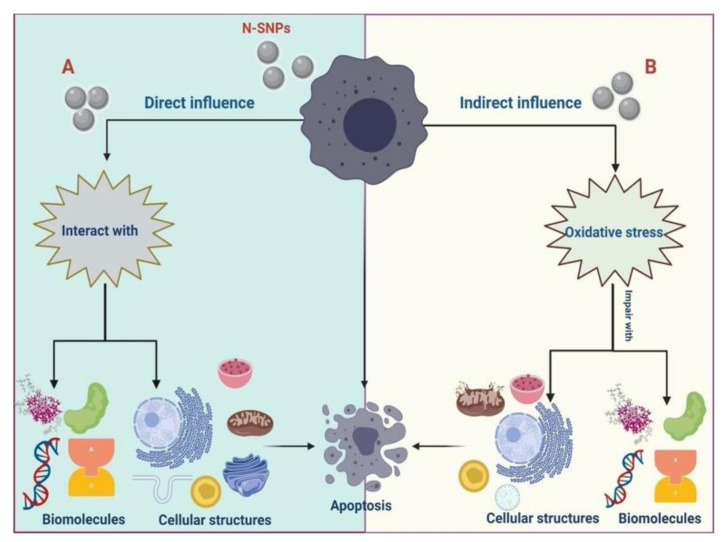
Possible strategies by which N-SNPs kill cancer cells.

We conclusively demonstrated the cytotoxicity of novel N-SNPs against MCF-7, HepG2, and HCT-116 cells by various experiments, including cell viability assays, inverted light microscopy and TEM, antioxidative marker and enzyme analysis, and western blotting assays. Morphometric measurements based on TEM micrographs were also used to detect changes in the number and size of cells and organelles after treatment with N-SNPs. In all tested cells, N-SNPs led to decreased cell proliferation, imbalances in GPx and GSH causing intensive oxidative stress, increased LDH leakage suggesting loss of membrane integrity, and a reduction in ATPase activity that results in metabolic toxicity. In addition, N-SNPs caused drastic cellular and subcellular alterations, demonstrating the ability of these NPs to induce cell death via interactions with cell membranes and organelles. N-SNP treatment also increased levels of antiapoptotic proteins (Akt, p-Akt, mTOR, and Bcl-2) and decreased levels of apoptotic proteins (p53 and caspase 3). The results indicate that the cytotoxicity of N-SNPs against the tested cells depends on two strategies: first, direct interactions with cellular components and organelles, resulting in cellular dysfunction and then cell death; and/or, second, an indirect influence through inducing oxidative stress, which induces cells to undergo apoptosis via effects on caspase 3 and p53 and the mTOR signaling pathway (Figure 13). The present findings are promising with respect to potential applications of N-SNPs as potent anticancer agents, as their ability to enhance oxidative stress and affect various cellular pathways leads to efficient apoptosis. Further studies are needed in order to explore the toxicity of N-SNPs against normal cell lines as well as their relationship with protein corona to test their selectivity and biocompatibility. Similarly, other studies are necessary to study anticancer activity of N-SNPs in vivo, alone or in combination with other therapeutic agents. The potentiality of these NPs to be functionalized with different therapeutic or functionable molecules for drug delivery or detection applications is needed to explore. More experiments are also required for elucidating the exact molecular pathways by which biogenic SNPs exert their effects on cancer cells. 

## Figures and Tables

**Table 1:** Antibodies used in the current study.

Antibody	Type	Product Code	Company
Akt	Rabbit monoclonal	EPR16798	Abcam
p-Akt	Rabbit polyclonal	ab8805	Abcam
Bcl-2	Rabbit polyclonal	ab59348	Abcam
mTOR	Rabbit monoclonal	ab32028	Abcam
p53	Rabbit polyclonal	ab131442	Abcam
Caspase 3	Rabbit polyclonal	ab13847	Abcam
β-actin	Rabbit polyclonal	ab228001	Abcam

**Table 2:** IC_25_ and IC_50_ values of *Nostoc*-mediated synthesized silver nanoparticles (N-SNPs) (µg/mL) used in all experiments against MCF-7, HCT-116, and HepG2 cells.

Cell	IC_25_	IC_50_
MCF-7	27 ± 0.02	54 ± 0.08
HCT-116	28 ± 0.04	56 ± 0.03
HepG2	40 ± 0.08	80 ± 0.02

## Supplementary Materials

The following are available online at https://www.mdpi.com/2072-6694/12/8/2099/s1, Figure S1: Effects of N-SNPs (IC25 and IC50) on antiapoptotic (Akt, p-Akt, Bcl-2, and mTOR) and apoptotic (p53 and caspase 3) protein expression levels in MCF-7, HCT-116, and HepG2 cells. Figures S2–S8: Uncropped images of the Western blots were utilized for the composition of Figure 11.

## Abbreviations

NPs	Nanoparticles
SNPs	Silver nanoparticles
nm	Nanometer
min	Minute
µL	Microliter
mg	Milligram
mL	Milliliter
µg	Microgram
mM	Millimolar
h	Hour
rpm	Revolutions per minute
kV	Kilovolt
kb	Kilobase
