# Supplementary material for: Oxidative Stress and Apoptotic Responses Elicited by Nostoc-Synthesized Silver Nanoparticles against Different Cancer Cell Lines
Source: Cancers (Basel). 2020 Jul 28;12(8):2099. doi: 10.3390/cancers12082099 (PMC7464693; doi:10.3390/cancers12082099)
Supplement: Supplementary file 1 [file cancers-12-02099-s001.pdf]

Supplemental Materials: Oxidative Stress and Apoptotic Responses Elicited by *Nostoc*-Synthesized Silver Nanoparticles Against Different Cancer Cell Lines

Reham Samir Hamida, Gadah Albasher and Mashael Mohammed Bin-Meferij

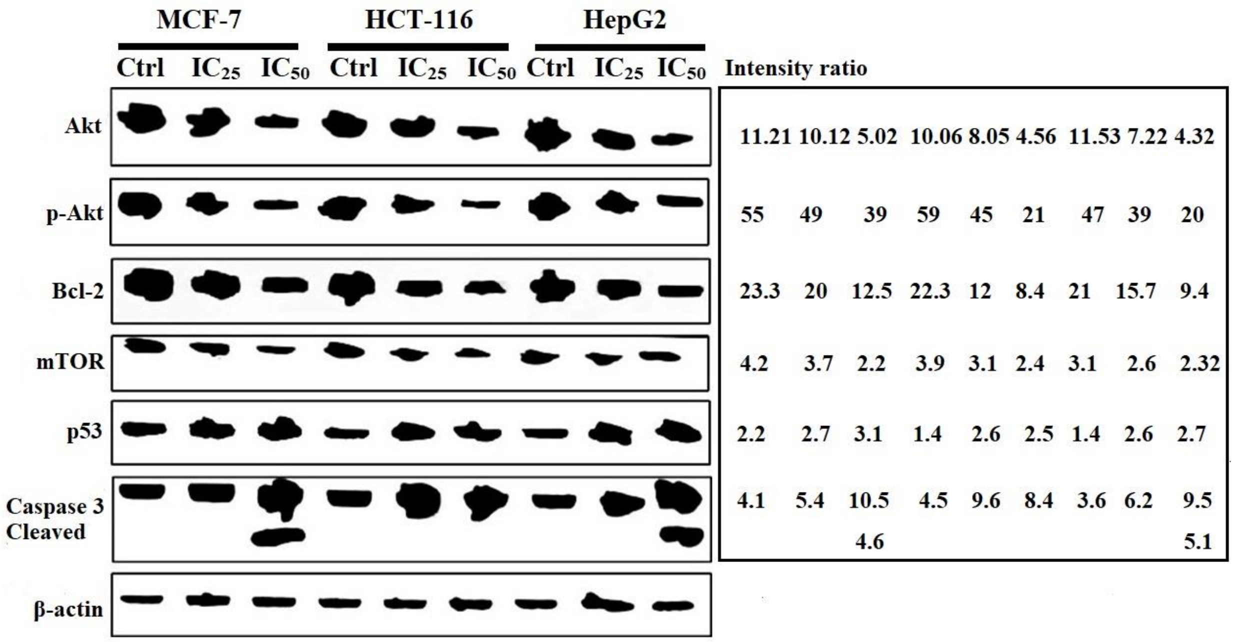

Figure S1. Effects of N-SNPs (IC_{25} and IC_{50}) on antiapoptotic (Akt, p-Akt, Bcl-2, and mTOR) and apoptotic (p53 and caspase 3) protein expression levels in MCF-7, HCT-116, and HepG2 cells. All of the data were collected from independent experiments performed in triplicate. The indicated ratios of band intensities are exhibited next the corresponding lane.

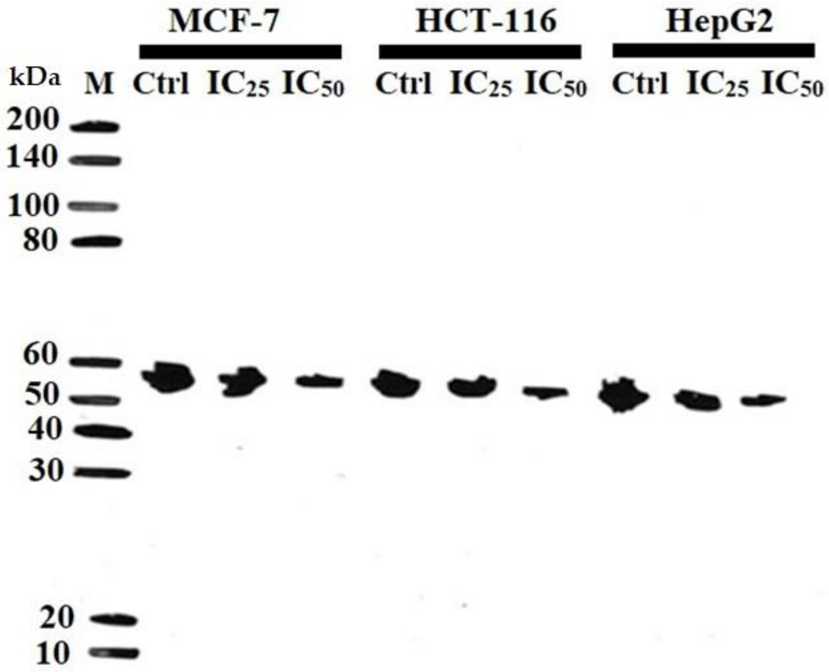

Figure S2. Uncropped image of Akt protein blot that was used for the composition of Figure 11.

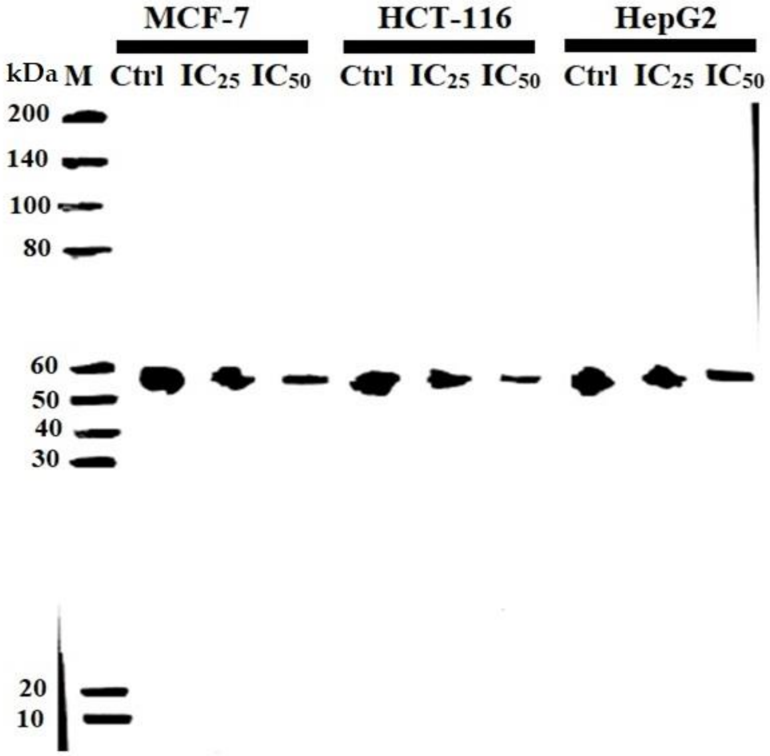

Figure S3. Uncropped image of p-Akt protein blot that was used for the composition of Figure 11.

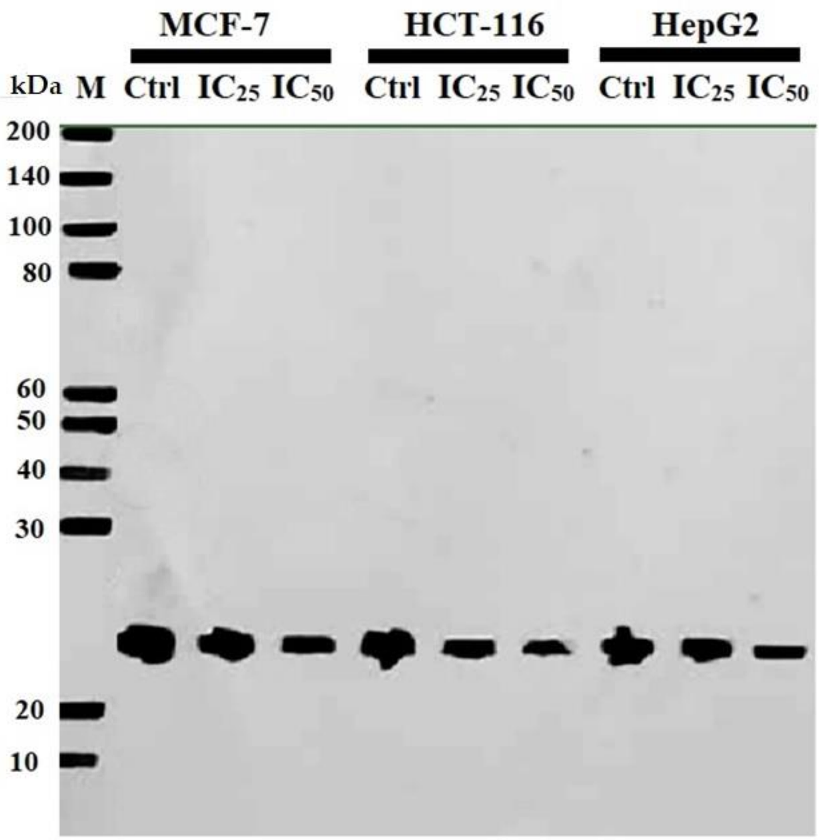

Figure S4. Uncropped image of bcl-2 protein blot that was used for the composition of Figure 11.

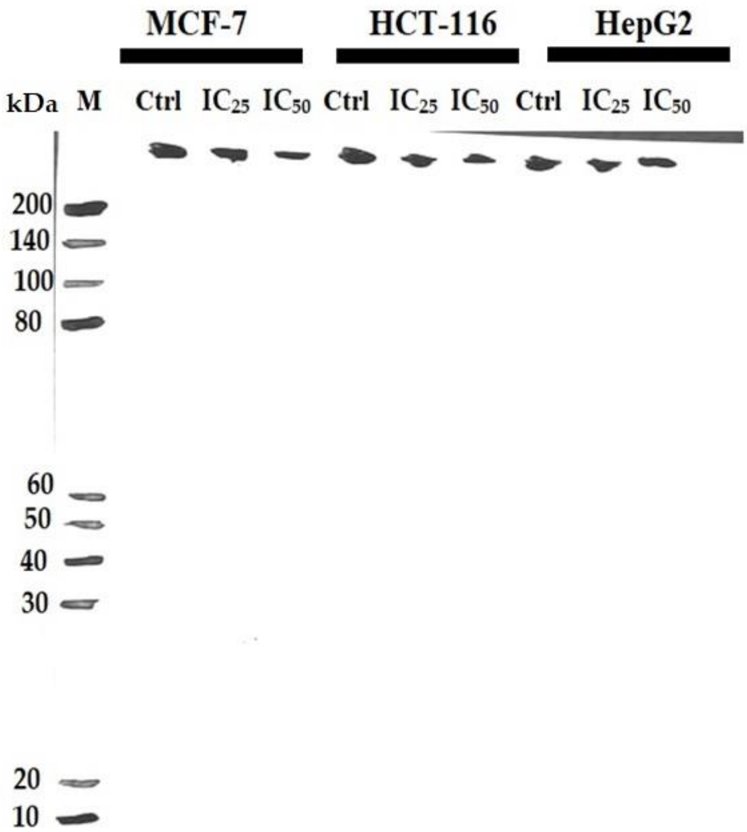

Figure S5. Uncropped image of mTOR protein blot that was used for the composition of Figure 11.

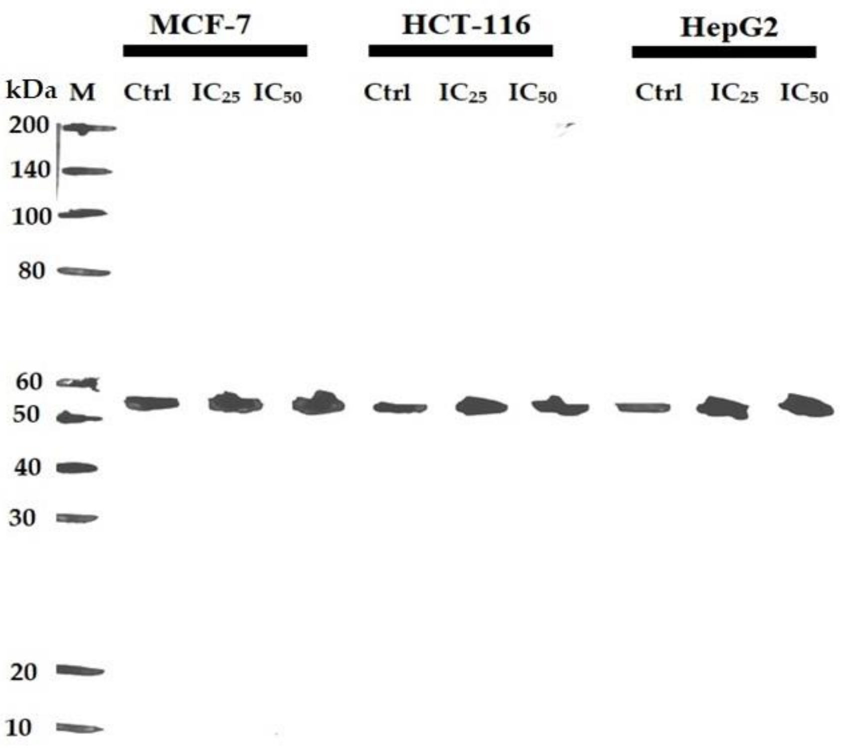

Figure S6. Uncropped image of p53 protein blot that was used for the composition of Figure 11.

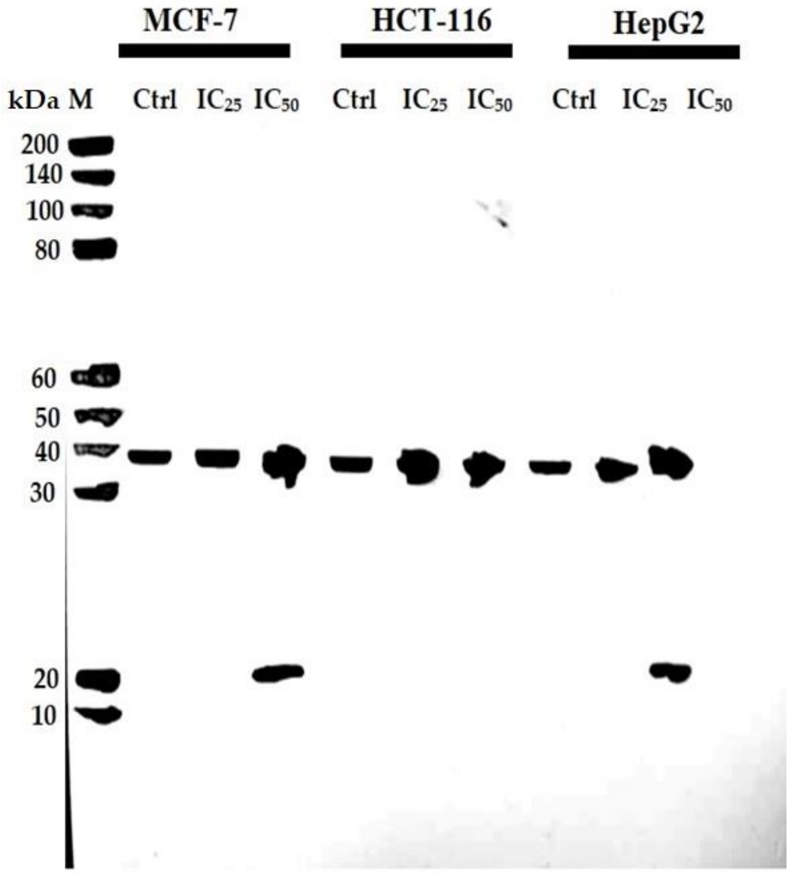

Figure S7. Uncropped image of caspase 3 protein blot that was used for the composition of Figure 11.

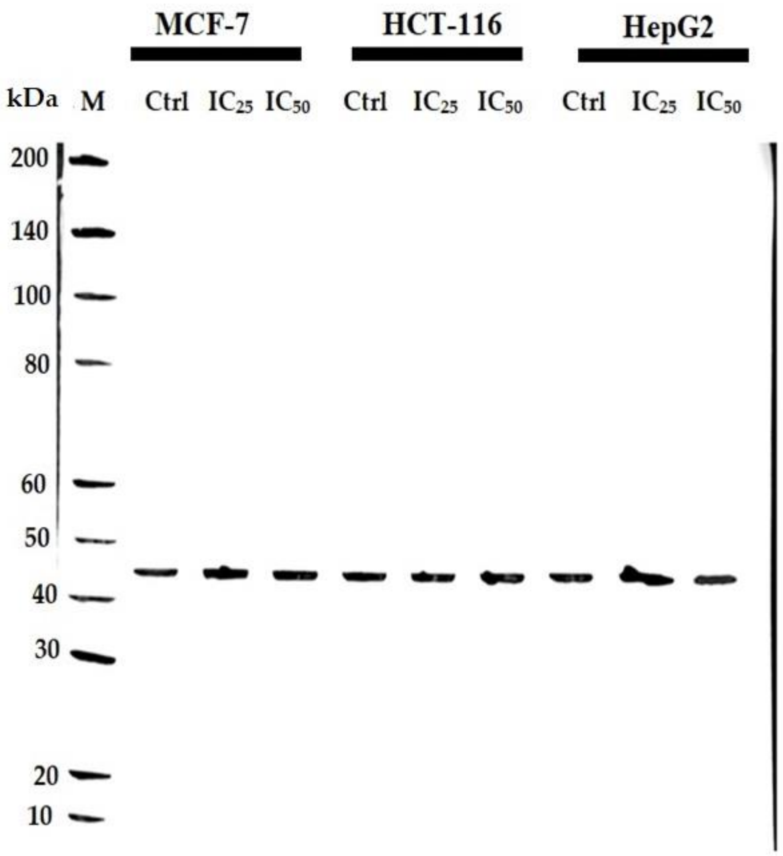

Figure S8. Uncropped image of β-actin protein blot that was used for the composition of Figure 11.

© 2020 by the authors. Licensee MDPI, Basel, Switzerland. This article is an open access article distributed under the terms and conditions of the Creative Commons Attribution (CC BY) license (http://creativecommons.org/licenses/by/4.0/).
